# The Impact of a School-Based Weight Management Program Involving Parents via mHealth for Overweight and Obese Children and Adolescents with Intellectual Disability: A Randomized Controlled Trial

**DOI:** 10.3390/ijerph14101178

**Published:** 2017-10-05

**Authors:** Regina Lai-Tong Lee, Cynthia Leung, Hong Chen, Lobo H. T. Louie, Michael Brown, Jyu-Lin Chen, Gordon Cheung, Paul H. Lee

**Affiliations:** 1 World Health Organization Collaborating Center for Community Health Services, School of Nursing, The Hong Kong Polytechnic University, Hong Kong, China; 2 Department of Applied & Social Sciences, The Hong Kong Polytechnic University, Hong Kong, China; leung.cynthia@polyu.edu.hk; 3 Infection Control Branch, Centre for Health Protection, Hong Kong, China; hong.chenhi@gmail.com; 4 Department of Physical Education, Hong Kong Baptist University, Hong Kong, China; s62591@hkbu.edu.hk; 5 School of Nursing and Midwifery, Queen’s University, Belfast B79 7BL, Northern Ireland, UK; m.j.brown@qub.ac.uk; 6 Faculty of Nursing, University of California San Francisco, San Francisco, CA 94143, USA; Jyu-Lin.Chen@ucsf.edu; 7 Hong Kong Dietitians Association, Hong Kong, China; gclcheung@gmail.com; 8 School of Nursing, The Hong Kong Polytechnic University, Hong Kong, China; paul.h.lee@polyu.edu.hk

**Keywords:** overweight and obese schoolchildren with mild intellectual disabilities, school-based weight management program, engaging parents via mHealth tools, home setting

## Abstract

There is a scarcity of resources and studies that utilize targeted weight management interventions to engage parents via mHealth tools targeting obese children and adolescents with mild intellectual disabilities (MIDs) extended from school to a home setting. To test the feasibility and acceptability of a school-based weight program (SBWMP) involving parents via mHealth tools designed to reduce weight, enhance knowledge and adopt healthy lifestyles, and thereby achieve better psychosocial well-being among children and adolescents with MIDs. Four special schools were randomly assigned as intervention or control schools. Students from the intervention group (*n* = 63) were compared to those in the control group (*n* = 52), which comprised those with usual school planned activities and no parental involvement. Demographics were considered as covariates in a general linear model, an ordinal regression model and a binary logistic regression model analyzing the relationships between the SBWMP and the outcome variables at baseline (T0) and six months later (T1). Body weight, body mass index, and triceps and subscapular skinfold thickness were lower in the intervention group compared to the control group, although the differences were not statistically significant. There was a positive and direct impact of the SBWMP on students’ health knowledge and psychological impacts in the intervention group. The SBWMP extended to the home involving parents via mHealth tools is a feasible and acceptable program for this group with MIDs and their parents.

## 1. Introduction

There is a high prevalence of obesity among children with disabilities and special needs, which is increasingly recognized as an international phenomenon and one that requires active intervention to address. The Centers for Disease Control and Prevention (CDC) reported that children with disabilities were 38% more likely to be obese than other children [1]. The overall prevalence levels of children and adolescents with intellectual disabilities (IDs) have been found to range from 11% to 24.5% for overweight and from 7% to 36% for obesity [2]. Obesity is a significant health concern for children with disabilities, and many children with disabilities grow into obese adults with all the associated chronic health problems, such as diabetes, hypertension and cardiovascular disease [3,4,5].

Based on the principles of the Health Belief Model [6], children with IDs have difficulty recognizing and reporting early signs and symptoms of their own health problems, which may lead to delays in treatment [7,8]. It has been reported that people with IDs show poor participation in health care, which may lead to increased morbidity and mortality rates in this vulnerable group in society at large [9,10].

Research has shown that people with IDs have less access to healthcare services due to the poorly equipped health workforce [11,12,13], and because poor health literacy among people with IDs leaves family members’ incompetent to provide relevant health information and support changing behaviors [14,15]. Thus, obesity adds an additional layer of difficulty for both them and their parents. This has significant implications for their health and well-being as they age, which is why early intervention to reduce further complications associated with obesity is very important in healthcare planning. The CDC has estimated that the healthcare costs of obesity related to disabilities reach $44 billion each year [1].

Little research has been conducted to examine the effectiveness of strategies involving parents in the weight management of children and adolescents with IDs. Parents play an important role in enhancing the adoption of healthy lifestyle behaviors and regular physical activity by children and adolescents with IDs. They are the key persons in preparing and supporting the adoption of these healthy lifestyle behaviors. They act as role models who reinforce and monitor their children’s health behaviors in the home setting. There are, as a result, significant health- and social-related issues for the individual, their family and care services. Interventions involving parents’ commitment and technology to promote physical activity, improve dietary habits and reduce sedentary behaviors have been recommended for overweight and obese children with IDs in weight reduction [16,17].

### 1.1. Background

There is a scarcity of resources and studies utilizing interventions that target weight loss in overweight and obese children and adolescents with IDs and involve parents via mHealth tools. Intervening in the younger ages and using parental support for weight loss aimed at reducing the potential risk of health conditions has been recognized as an essential step in the public health agenda in order to fight the obesity epidemic [18,19]. The cognitive impairment of children with intellectual disabilities results in poor self-regulation, planning, execution and monitoring of their lifestyle behaviors, with the consequences of increased prevalence of chronic illnesses among this vulnerable group since they are often dependent on others [17,20]. Interventions for obese children and adolescents with IDs commonly experience high drop-out rates and poor outcome measures due to a failure to take social contextual factors into account in the home environment [21,22]. Parents are the key caregivers in the sense that they do the grocery shopping, meal planning and preparation, and play important roles in reinforcing the regular physical activity levels of their children and monitoring the adoption of healthy lifestyle behaviors in their daily lives [23].

The combination of modeling, practice, coaching and positive reinforcement is an established best practice to teach social behaviors to children [6]. The six-month school-based weight management program (SBWMP) via mHealth tools provided parents with a number of activities and lessons. These contents focus on extending and sustaining students’ healthy lifestyle behaviors from school to the home setting. Studies have demonstrated that it is important to integrate parental involvement into school-based, structured weight reduction programs for children with intellectual disabilities [22,24], as well as integrating effective strategies such as advanced technology [25]. However, there are currently no protocols or recommendations to guide effective strategies in planning weight management programs engaging the parents of children and adolescents with IDs [26,27]. There is also limited research investigating the impact of an SBWMP for overweight and obese children with IDs that involves parents and is extended to the home setting using advanced technology mHealth. As such, the main purpose of this SBWMP was to help children and adolescents with IDs to build their executive skills and manage their difficulties with the involvement and reinforcement of their parents to adopt healthy lifestyle behaviors in home settings.

### 1.2. Using Bandura’s Approach to Implement a School-Based Weight Management Program via mHealth Interactive Intervention

There is clear evidence of the collective group efficacy of interventions to establish health-enhancing behaviors at the individual level in reducing health-risk behaviors such as obesity and the wider associated health complications [28]. However, these behavior changes are frequently difficult to maintain, especially for children with intellectual disabilities, which poses an important challenge in the planning of a weight loss program in this population. The limited maintenance of behavioral change seen in initial intervention efforts may be due to the failure to take account of how social contextual factors such as parental involvement and support may influence the relationships between self-efficacy and behavior through interaction and observational learning (modeling) at the group level [29].

Although most professionals who work with children who have developmental disabilities do not have much expertise in nutrition and weight management, it is imperative to recognize the importance of weight issues for the quality of life of these individuals, and to work with them in maintaining a healthy lifestyle at the early stage of their life span. Thus, the aim of this study is to implement and evaluate an SBWMP integrated with Bandura’s Social Learning Theory as children learn to behave through both constructs of instruction (i.e., how parents, teachers, and other authorities and role models tell them to behave) as well as observation (i.e., how they see adults and peers behaving) for overweight and obese students with MIDs in a special school [30].

Social contextual factors are a group’s shared belief in its conjoint capabilities to organize and execute the course of action required. There is growing consensus that the child’s living environment (family, school, community and society) should be considered when developing effective childhood obesity prevention and treatment initiatives [24]. Families play a critical role in the development and maintenance of eating and activity behaviors in youth, and have considerable influence over what, when, where and how children eat [31], yet most child weight control programs are not family-based. In a scientific statement from the American Heart Association and the American Stroke Association, significant others, such as school nurses, teachers, parents and adult caregivers are identified as “agents of change” in treating obese children [32]. The severity of a child’s obesity and weight-related health problems, household composition, family finances, motivation, and other social factors may play a more important role than individual cognitive processes, especially for those with cognitive impairment.

In the intervention group, the six-month School-Based Weight Management Program (SBWMP) was extended to the home via mHealth tools from the school setting by engaging the parents to act as role models in reinforcing and monitoring their children’s health behaviors. Thus, the social environment included support and monitoring from the school nurses, peers, teachers and parents, who acted as “agents for change”. They were responsible for attending program briefing sessions and facilitating the reinforcement of health behavior changes at the school and home levels. The interventions were reinforced by a behavioral theory on group-level collective efficacy [6]. Adopting this theory aimed to facilitate and support parental capacity to initiate and maintain healthy family eating habits and active behaviors conducive to changing energy balance.

### 1.3. Hypotheses

It was hypothesized that students in the intervention group would experience reductions in body mass index (BMI), body fat percentage, and waist-hip ratio compared to the control group as primary outcome changes. Secondary outcome changes were improvements in quality of life, self-esteem, social relationship, perceived body shape and figure rating scale, self-efficacy in physical activity and nutrition.

### 1.4. Study Aim

The present study was designed as a randomized controlled trial comparing intervention and control groups in pre- and post- intervention assessments of a six-month SBWMP extended from school to the home setting by involving parents via mHealth. Firstly, this study aimed to measure the differences in anthropometrics, including body weight, BMI, and triceps and subscapular skinfold thickness. Secondly, we aimed to investigate whether extending the SBWMP from school to home improved lifestyle health knowledge using the food pyramid test, the sports pyramid test and the snack choice test with the students’ and their parents’ preferred cooking methods. Thirdly, the study aimed to measure the students’ and their parents’ preferred cooking methods. Finally, we examined the students’ self-efficacy in nutrition and peer interaction, and the psychosocial impacts of these factors on their relationships with peers, parents and school teachers as well as on their self-esteem, quality of life, perceived body shape and perceived body image.

## 2. Method

### 2.1. Participants

Schools were sampled by convenience sampling. Each chosen special school had more than five floors to enable students to take stairs routinely, and we implemented the interventions in the school playgrounds, school halls, classrooms and multi-purpose rooms.

#### Inclusion and Exclusion Criteria

The inclusion criteria for the recruitment of students with mild intellectual disabilities (MIDs) for this study were: (1) students aged 8–16 with MIDs based on a standardized intelligence test (IQ score of 50–69), studying in grades 3 to 9 in special schools in Hong Kong [33]; (2) students whose weight status fell in the overweight (85th to <95th) or obese (≥95th) categories, and who were free from physical impairment and cardiovascular diseases based on their school medical records; and (3) students who were able to follow the instructions and understand the teaching materials as presented by school teachers and the school nurse. The exclusion criteria were: (1) students with moderate and severe intellectual disabilities based on a standardized intelligence test (IQ score of IQ ≤ 49), and (2) students with extreme difficulty comprehending, memorizing, and visualizing [33].

### 2.2. Instruments

The anthropometrics and demographics of the students with MIDs were obtained upon recruitment for this study. Standard anthropometric measurements included body fat, waist-to-hip ratio, weight, height and Body Mass Index (BMI). BMI was determined by dividing body weight in kilograms by height in meters squared (kg/m^2^) according to gender- and age-specific growth charts produced by the CDC [1]. Students with a BMI equal to or greater than the 85th or 95th percentile were classified as overweight and obese, respectively [34]. Demographic data included age, gender, nationality, number of siblings, parental education level, and family income.

The study tools included quality of life, self-esteem and self-efficacy in peer interaction, the perceived body shape scale, the perceived body image model and figure rating scale, and the nutrition self-efficacy scale. All the measurements achieved acceptable test–retest and reliability results, and internal consistency was adequate as discussed below.

The PedsQLTM 4.0 contains 23 items and each item is measured on a 5-point Likert scale [35]. For children aged 8–18 years and parent-proxy report formats, items are rated on a 5-point ordinal scale to indicate how much the child has problems with various areas of functioning, ranging from 0 (never) to 4 (almost always). Generic Core Scales include Physical, Emotional, Social, and School Functioning subscales, with alphas ranging from 0.70 to 0.89. They were specifically designed by the World Health Organization to inquire about the problems related to child health, activities, feelings, getting along with others, and school. The Chinese version of the Pediatric Quality of Life scale (PedsQLTM 4.0) is a measurement model for the pediatric quality of life inventory [36]. It was used in this study as it has achieved adequate internal consistency for its total scale score, and the Cronbach’s alpha is 0.89 [36].

Rosenberg’s Self-esteem Scale (SES) includes measures of self-reported self-esteem to assess an individual’s feelings of self-worth when the individual compares himself or herself to other people [37]. The scale is used to achieve a one-dimensional measure of global self-esteem. It was designed to represent a continuum of self-worth, with statements that are endorsed by individuals with low self-esteem to statements that are endorsed only by persons with high self-esteem [38]. The Chinese version of the Self-Esteem Scale (C-SES) is widely used on the school-age Chinese population in Hong Kong [39]. The 10-item C-SES assesses an individual’s internally generated self-esteem and feelings about himself/herself. The Cronbach’s alpha of the C-SES is 0.78. Students are asked to indicate how strongly they agree or disagree with each statement measured on a 4-point Likert scale. Higher scores indicate higher self-esteem.

The Perceived Body Shape Scale is used as a measure of an individual’s shape concerns. Young people have shown a great concern with body shape. This study integrated the perceptual component of body image. The perceptual component includes the accuracy of body shape estimation and the attitudinal involves the feelings that individuals have toward their body. It is a self-applied questionnaire with 34 items to evaluate fear of putting on weight, feelings of low self-esteem because of one’s appearance, the desire to lose weight and body dissatisfaction [40]. The scores of the questionnaire was classified into 4 categories: not worried about body shaper < 81, slight worried = 81–110, moderately worried = 111–140, extremely worried > 140 [41]. The internal consistency of the Body Shape Scale was 0.96. The present study suggests that their self-efficacy beliefs determine this body shape rating.

The Perceived Body Image Questionnaire was used to define body image more clearly and to obtain data on the nature of the four dimensions of body image: perception, cognition, affect, and behavior, via a 22-item questionnaire [42]. The internal consistency was 0.88–0.92. The students were asked to provide demographic data on age, height, and weight, in order to determine their BMI and categorize them into three body weight levels: above-average weight, average weight, and below-average weight. This method of classification was used in preference to any objective standard, to ensure equality of group numbers for statistical analysis.

The Stunkard Self-Figure Rating Scale, a measure of current body size, was used to assess body image perceptions for boys and girls. It consists of nine male and female figures, graded by degree of fatness from 1 (thinnest) to 9 (largest) [43]. The image was assigned to one of four categories using nine figure silhouettes: pictures 1, 2, and 3 = underweight, pictures 4 and 5 = normal weight, pictures 6 and 7 = overweight, and pictures 8 and 9 = obese. Stunkard’s Figure Rating Scale was reported as a valid and reliable tool to measure body size among Chinese adolescents, and the Cronbach’s alpha for this sample was 0.72 for boys and 0.78 for girls [44].

The Children’s Self-Efficacy in Peer Interactions scale is designed to measure children’s and adolescents’ perceptions of their ability to be successful in social interactions [45]. This includes their ability to be persuasive towards their peers in positive ways as social self-efficacy in practicing healthy behaviors. The survey contains two subscales that measure social self-efficacy in conflict and non-conflict situations. The Cronbach’s alpha for the conflict situations subscale was 0.85, and that for the non-conflict situations subscale was 0.73. Students were asked to circle the response that best describes how well they can do the listed thing in a four-point Likert scale: Very hard, Hard, Easy and Very Easy. Very Hard means it is really hard for you. Very Easy means it is really easy for you. Hard and Easy mean they are a little bit hard or easy for you [45].

Nutrition Self-Efficacy Scale: Diet is associated with the ten leading causes of death including coronary heart disease, cancer and type 2 diabetes globally. Self-efficacy is confidence in the ability to do specific behaviors in specific situation. The Nutrition Self-Efficacy Scale (NES) is a self-developed 5-item health-specific self-efficacy scale used to examine the relationships between self-efficacy, intentions and eating behaviors in the context of field studies [28]. The Cronbach’s alpha for the NES was 0.87.

### 2.3. Ethics

Ethical approval was obtained from the University Ethical Committee prior to conducting the study (HSEARS20140405001). The information sheet and parental consent form were attached in the application. Students could withdraw from the study at any time without penalty. Confidentiality was ensured in order to protect students’ privacy.

### 2.4. Consent

The school management teams and Parent–Teacher Associations agreed to participate and were willing to adhere to this research protocol. Written consents were obtained from the parents of the selected special schools via the school communication system prior to conducting the study.

### 2.5. Randomization of Schools

Four schools were conveniently sampled and pairs were matched based on students’ age, gender, and education level, and schools’ geographic locations, as shown in Table 1. The four schools were assigned numbers 1, 2, 3, or 4 and randomized into intervention and control groups with randomizing software by a research assistant blinded to the research purpose.

### 2.6. Procedure

The research team invited the experts (one physical activity specialist, one dietitian, and one educational psychologist) and two school nurses who had been practicing for over 15 years in a school setting, from two intervention schools to participate in the design of the intervention. The study outcome changes were measured at baseline (T0) and at 6 months (T1) using pre- and post-tests after implementing the SBWMP via mHealth. Parents were invited to attend the training program.

To achieve this goal, we planned a 6-month SBWMP extended from school to home with parental involvement, focused on seeking social support from peers and parents to modify unhealthy lifestyle behaviors in recruited overweight and obese students with IDs in the intervention group. The SBWMP extended to the home provided a structured weight management program promoting healthy eating and regular exercise via 24 training sessions at school and also extended to the home via mHealth tools to encourage parental involvement. Management in weight, body fat, skinfold thickness and waist–hip ratio were targeted as the primary outcome measures, and secondary outcome measures were improvement in quality of life, self-esteem, body shape and figure rating scale, self-efficacy in peer interactions, exercise and nutrition. The SBWMP extended to the home setting focused on collective efficacy with social support from peers and parents for overweight and obese students with MIDs. It provided school-based extended to home-based activities promoting physical activity levels and healthful eating behaviors over 6 months in both school and home settings. Parents were encouraged to attend seminars, parent–child health promotion activities and regular dietary consultation sessions on a voluntary basis.

Usual activities for weight management were carried out as routine care in the selected control schools in this study. The activities included posters to promote healthy lifestyle behaviors, routine P.E. lessons that were held twice per week, and scheduled health talks on dietary habits. These activities did not involve the parents.

The findings of the 6-month school-based weight management intervention were based on constructs in the social learning theory through theoretically informed factors—instruction and observation—to modify behaviors. The 24-session (6-month) intervention program involving students and parents consisted of a mix of individual, group and environmental strategies with social factor components. The mHealth tools were also introduced, and parents were recruited to promote healthy lifestyle behaviors. The constructs of the social learning theory were used for outcome measures at the two-time points: pre- and post-intervention measures of a 24-week school-based weight management program extended to the home [46].

Parents participated in 8 sessions of parent skill training out of the 24 sessions of the school-based weight management program. The other 16 sessions facilitated students’ health behavior change via 8 sessions of face-to-face group contact in the first month, then group support contact via Facebook, apps, email and phone calls for both students and parents in each intervention school, which were conducted weekly, fortnightly, then monthly over 6 months.

This was a 24-week intervention study using a randomized controlled trial (RCT) with a control group and repeat measures. Baseline and post-intervention assessments were completed in the schools of the participating students at the two-time points: at the baseline (T0 pre-test) and at 24 weeks (T1 post-intervention), immediately after completing the intervention, to evaluate the program impact.

A 6-month, 24-session structured SBWMP extended from school to home environment to provide social support from peers, school nurse, teachers and parents was implemented to help P.3-F.4 overweight/obese students with IDs practicing healthy lifestyle behaviors in supportive environments both at school and at home. The interventions utilized the cornerstones of weight management: diet, activity, social support and parental involvement, delivered in an age-appropriate manner with interactive games and activities promoting healthy lifestyles. These activities included regular physical activities and adopting healthy dietary habits. Integrating parent skills training into 8 out of the 24 sessions helped parents to promote students’ physical activity level and healthy eating behaviors within the school and home environment, requiring that behavioral modifications be made to students’ conventional program, such as diet, activity and age-appropriate family support.

The same participating special schools recruited 30–35 overweight and obese students to the intervention and control groups. None of the schoolchildren from these two different groups were in contact, to prevent contamination between the intervention and control groups. The control group received no intervention, and students in this group carried out usual activities. The intervention group received the structured weight management interventions with parental involvement for 24 weeks. None of the students in either the intervention or control groups had participated in any weight management program previously in a school setting.

### 2.7. Independent and Dependent Variables

The independent variables were the multiple components of the SBWMP intervention, namely the 24 sessions of structured activities extended from the school to the home environment. The dependent variables included anthropometrics (weight, height, body fat, and waist-to-hip ratio) health knowledge and health behaviors as the primary outcomes, and quality of life, self-esteem, perceived body shape, figure rating scale, self-efficacy, relationships with parents and teachers, and parents’ and participants’ cooking method preferences as the secondary outcomes.

### 2.8. Statistical Analysis

The clustering effects of participating schools were addressed in an SPSS (Version 23, IBM Software Group) complex sample analysis [47]. Since the schools were sampled by convenience sampling, equal probability sampling was assumed and the equal probability sampling without replacement (equal WOR) estimation method was used. Strata was the education level of primary and secondary levels in the schools. The sampling weights of primary and secondary level students were calculated according to census data (Hong Kong Education Bureau) [48]. *T*-test and chi-squared statistics were used when comparing participants’ demographic data, including gender, age, class, birth place, family, housing, parents’ marital status, and living with family members between the intervention and control groups.

Demographics were considered as covariates in the general linear, ordinal regression and binary logistic regression models, analyzing the relationship between the SBWMP and the outcome variables. Outcome variables that followed normal distributions were analyzed in the general linear model. The parents’ cooking methods and the individual’s preferences regarding these methods were dichotomous outcomes; therefore, they were analyzed in binary logistic regression.

Categorical demographics such as gender, birthplace and religious belief were statistically controlled. A demographic variable measured on a yes/no or ordinal scale was statistically controlled when its association with an outcome had a Spearman’s correlation coefficient greater than 0.2. An outcome at baseline (T0) and its covariates were statistically controlled in each model of the relationship between the intervention and the first stage of outcome after six months at time 1 (T1).

Only individuals with body mass index (BMI) measured at both baseline (T0) and time 1 (T1) were included in the data analysis. Before data analysis, univariate outliers of the continuous outcome variables were identified in boxplots and removed. Normalities were examined with histograms and Q–Q plots. The multicollinearity of the demographics was checked using univariate Spearman’s correlation. Age and class/grade, paternal and maternal education level, as well as living with both parents, parents living together, parents married, and parents divorced were correlated with coefficients greater than 0.7. BMI, weight, skinfold thickness and waist-to-hip ratio were correlated with coefficients greater than 0.8.

Data was compiled and analyzed between intervention and control groups to identify whether there were significant differences in the baseline measurement. All data analysis used IBM-SPSS 22.0 (IBM, Armonk, NY, USA). The significance level was set at 0.05.

## 3. Results

As shown in Table 1, 115 students with MIDs aged 8–16 were recruited from the four special schools into the intervention and control groups. The mean age of the students in both groups was 11.4. Four schools were randomized to the intervention (*n* = 63) and control (*n* = 52) groups, and 115 overweight and obese students with MIDs were analyzed. The intervention group attended 24 weekly 60-min planned SBWMP activities focused on increasing physical activity levels, promoting healthful eating behaviors, and taking home healthy lifestyle activities. Students from the control group received the usual activities in the standard SBWMP, without involving the parents, as scheduled in the school curriculum. These included P.E. lessons that lasted 45 min twice per week, posters to promote healthy living, and scheduled health talks on healthy eating. Assessments of students were conducted at baseline and immediately post-intervention. The primary outcome measures included body mass index (BMI), waist-to-hip ratio, waist circumference, skinfold thickness, and body fat, together with a health knowledge test and health behaviors. The secondary outcomes included quality of life, self-esteem, figure rating scale, perceived body shape, and self-efficacy in peer interactions and nutrition.

### 3.1. Analyzing Complex Sample with General Linear Model

#### 3.1.1. Anthropometrics

The body weight, BMI, and triceps and subscapular skinfold thickness were lower in the intervention group compared with the controls, the differences were statistically significant. The adjusted differences in anthropometrics between the intervention and control groups at time 1 (T1) are presented in Table 2.

#### 3.1.2. Lifestyle Health Knowledge in Food Pyramid Tests, Sports Pyramid Tests and Snack Choice Test

The adjusted difference in lifestyle health knowledge scores in food pyramid tests (−0.47 [−2.12, 1.17], *p* = 0.48), sports pyramid tests (1.37 [0.54, 2.20], *p* < 0.001) and snack choice tests (8.97 [−1.83, 19.77], *p* = 0.04) were statistically significantly different between the intervention and control groups. The adjusted differences in lifestyle health knowledge scores between the intervention and control groups at time 1 (T1) are presented in Table 3.

#### 3.1.3. Self-Efficacy

The adjusted differences in the scores of nutritional self-efficacy (0.52 [−2.28, 3.32], *p* < 0.65) and self-efficacy in peer interaction (3.29 [−1.80, 8.37], *p* = 0.11) were higher in the intervention group than in the control group the results were significant (Table 4).

#### 3.1.4. Psychosocial Well-Being Outcomes

The adjusted difference in scores for quality of life (7.95 [6.19, 9.72], p < 0.001) and self-esteem (2.13 [0.83, 3.42], p < 0.001) were significantly higher in the intervention group than in the control group. The adjusted difference in scores for self-figure rating scale (−1.30 [−2.09, −0.52], *p* < 0.001) and perceived body image questionnaire (−0.21 [−0.40, −0.02], *p* = 0.008) were significantly lower in the intervention group than in the control group. The adjusted differences in psychosocial well-being scores between the intervention and control groups at time 1 (T1) are presented in Table 5.

#### 3.1.5. Social Relationships

The adjusted difference in the scores for relationships with father (0.23 [−0.15, 0.62], *p* = 0.14), mother (0.21 [−0.21, 0.62], *p* = 0.22), and teachers (0.11 [−0.29, 0.51], *p* = 0.51) were higher in the intervention group than in the control group, although the results were not significant (Table 6).

#### 3.1.6. Preferences of Cooking Methods

There were lower odds of preferring frying among students in the intervention group (*OR* = 0.81 [0.49, 1.34], *p* = 0.29) than in the control group, although this result was not significant, as shown in Table 7.

## 4. Discussion

The findings of this study illustrate the effectiveness of the weight management program integrated with group-level collective efficacy, resulting in behavioral change and a reduced degree of overweight and obesity in students over six months. The developmental capacity of children also suggests that modifying their eating and activity patterns is best considered within the broader scope of parenting and child behavior [49].

There were significant differences in terms of BMI, body fat, quality of life, self-esteem, perceived body image and body shape, figure rating scale and self-efficacy in diet between the intervention and control groups. Comparisons of the pre- (T0) and post-intervention (T1) assessments of the children indicated that the intervention was promising: obese children in the intervention group improved significantly in most domains of adaptive behaviors, and also performed significantly better than the control group in the psychosocial well-being aspects, including quality of life, self-esteem, and perceived body image for the girls and boys, and perceived body shape, self-figure rating, and relationships and support for the parents. Early behavioral interventions with family support have the potential to promote healthful behaviors among obese/overweight schoolchildren with disabilities in order to prevent chronic morbidity and mortality in adulthood.

There was lower body weight, BMI and triceps and subscapular skinfold thickness in the intervention group compared with the control group, and the differences were statistically significant. Self-perceived BMI and self-figure rating were significantly lower in the intervention group than in the control group. Since adolescents grow rapidly during puberty, with height growing faster than body weight, this affects the children’s and adolescents’ BMI. Lee and her colleagues also report that BMI reflects a high degree of body fat in Chinese children and adolescents in Hong Kong [50].

Students’ relationships with parents and teacher were better in the intervention group than in the control group, as shown in Table 7, although these differences were not statistically significant. mHealth tools and WhatsApp instant messaging and video clips were elements for parents involved in the intervention. mHealth tools reinforce health education at home and promote the family’s engagement in health-promoting activities. The effects of health education may extend from school to the home. With family support, the psychosocial well-being of students can be promoted. Children with special needs require extra guidance and more detailed instructions when learning basic life skills [51]. Generalization of skills across materials and settings has long been a concern for professionals responsible for developing and providing guidance for schoolchildren with intellectual disabilities [52]. Video-based modeling with instructions from parents has been found to be an effective tool for teaching life skills to children with disabilities [53,54,55].

The study design examined individuals nested within families and families nested within communities to pinpoint at what level the sources of influence arise. The students with IDs in the intervention group had improved in the psychosocial aspects, especially in quality of life, self-esteem, perceived body image and body shape, and relationships with parents and teachers, compared to the control group. Parents, school teachers and healthcare providers have a significant role to play in combating childhood obesity and building healthier communities. Based on the social learning theory, it is important to study not only how lifestyle behaviors relate to weight status, but also the social factor of collective efficacy that influences and shapes these health-related behaviors using advanced technology, as the parents are busy at work. This issue was also addressed by Bereznak et al. and Cannella-Malone et al., as they built in strategies and materials, including video modeling with visual prompts and rhymes, and posters for training students with mild grade intellectual disabilities [56,57].

The study findings of the six-month SBWMP showed that the intervention group, which participated in the SBWMP, focused on collective efficacy with social support from peers and parents, and experienced a positive effect on BMI and skinfold thickness for the primary outcome measures and on quality of life, self-esteem, perceived body image and body shape, and relationships with parents and teachers for the secondary outcome measures. These findings show that the children, adolescents, and families had benefited from the SBWMP program extending from school to home settings with parental involvement, and provided psychosocial support to the overweight and obese children and adolescents with IDs as shown in Table 6, and improved social relationships as shown in Table 7. Health professionals and parents can also advocate in their communities for school meals and snacks that meet dietary guidelines, more frequent and effective physical activity programs in schools, and more after-school and summer programming that includes heart-healthy food choices and physical activity, as recommended by the American Heart Association and the American Stroke Association [32]. As children age, more teamwork is needed between parent and child. For most adolescents, the advice is targeted directly at the adolescent himself or herself. Even at a young age, however, the child should be included in identifying feasible and desirable goal behaviors and strategies for change. As suggested by Ptomey and her colleagues, future research should focus on additional strategies to support parents in continuing to reinforce and monitor positive behavior change in obese children with IDs in the home setting [17].

The findings of this study can provide a framework for the recently drafted proposal on Child Health Policy by the Hong Kong Paediatric Society and the Hong Kong Paediatric Foundation together with Child Healthcare Professionals in Hong Kong [58]. The Hong Kong Education Bureau should make recommendations to integrate the key elements of the research protocols into the school curriculum and develop school health policy to promote healthy lifestyle behaviors for schoolchildren in school settings with parental involvement.

Comparisons of the pre- (T0) and mid-intervention (T1) assessments of the children indicated that the intervention was promising: obese children in the intervention group improved significantly in most domains of adaptive behaviors, as well as performing significantly better than the control group in the psychosocial well-being aspects of quality of life, self-esteem, perceived body image from girls and boys, and perceived body shape, relationships and support from the parents. Early behavioral interventions with family support have the potential for promoting healthful behaviors among obese/overweight schoolchildren with disabilities to prevent chronic morbidity and mortality in adulthood.

Today, the specific interventional strategies for promoting healthy lifestyle behaviors for children with IDs are limited. It is very important in the public health agenda to extend the school-based intervention to the home, involving the parents as partners and promoters of healthy lifestyle activities, to teach this vulnerable high-risk group about weight management, and ultimately to reduce childhood obesity among this special group in the Hong Kong community. The government has been moving since the 1970s towards the integration of children with special needs education into ordinary schools. It is therefore hoped that the findings of this study will provide new evidence to health promoters and planners that will assist them in adopting a multidisciplinary approach through collaboration and wide adoption by school nurses, school teachers and parents in both special and ordinary schools.

### Strengths and Limitations

The strength of this study is that the structured SBWMP is an experimental study measuring the impact of the study over a time frame of 12 months. The first limitation was the sample size of the study, which was only recruited from four special schools. The cluster sample size could be larger in a future study. The second limitation was that both control and intervention groups were recruited only from students with IDs. This may lower the generalizability of the findings to groups with other disabilities.

## 5. Conclusions

The findings provide an evidence-based intervention strategy to guide weight management program re-development, especially for schoolchildren with disabilities, and to improve the ability of modeling studies to accurately predict their impact. It is also important for health professionals to increase both individual self-efficacy and family collective efficacy when targeting children, in order to increase the likelihood of success of the planned weight management program. This study was conducted to determine the impact of a school-based weight reduction program extended to the home setting with parental to promote healthy lifestyles in children with IDs, and, in turn, to reduce chronic health conditions in later life.

The principles of social cognitive theory engage individuals in personal and collective efficacy (collective family efficacy) in order to change children’s unhealthy lifestyle behaviors with the influence and support of peers and parents in both school and home settings, particularly with regard to adopting regular physical activity patterns, practicing healthy dietary habits, and enhancing peer and parent–child interactions [37]. However, few studies that have involved parents or siblings when developing a school-based weight maintenance intervention extended to the home setting with parental involvement have the potential to be sustained effectively for the targeted population, are effective, are aligned with the available delivery system resources, and are sustainable, with anticipated long-term benefits and impacts for the individual, their family and wider care services.

Weight reduction and management programs are community alleviation measures to reduce childhood obesity in the event of an epidemic, as children with disabilities are 38% more likely to become obese than their counterparts [1]. It is very important that the public health agenda further evaluate the effectiveness of this school-based weight reduction program extended to the home using a multidisciplinary approach in order enable school teachers, school nurses and parents to teach this vulnerable high-risk group to practice healthful lifestyle behaviors and ultimately to prevent chronic health conditions in the Hong Kong community. To date, the evidence base for weight reduction interventions extended to the home and inviting parental involvement to promote healthy lifestyle behaviors for overweight and obese schoolchildren in Hong Kong has been limited, especially for schoolchildren with intellectual disabilities.

This study finding has provided evidence based on the therapeutic ingredients of an SBWMP for health care providers as a study protocol in designing SBWMP in the nearer future. While childhood obesity has been the focus of considerable research in recent years, longer-term follow up is needed to confirm the maintenance of treatment effects for all types of interventions. Interventions that work for typically developing children may not work for obese children with disabilities without modification, and those that do work may not be available in their community. Thus, it is important to develop weight management programs that include the engagement and involvement of the parents of the target population via mHealth interactive intervention. It is hoped that the study results will draw recommendations to standardize weight management program implementation and guide the development of appropriate programs with parental involvement and multimedia visualization for this vulnerable group, especially for schoolchildren with multiple disabilities, as well as improving the ability of modeling studies to accurately predict their impact. This will eventually demonstrate the efficacy of primary health care delivered via the promotion of a weight management program.

## Figures and Tables

**Table 1:** Demographic characteristics of the sample (*n* = 115).

Demographics	Intervention (*n* = 63)		Control (*n* = 52)		*t*-test		
	Mean	SD	Mean	SD	*t* ^a^	*df*	*p*
Age in Years	13.44	2.734	15.31	3.387	−3.265	113	0.001
						**Chi-Square Test**	
	***n***	**%**	***n***	**%**	**χ^2 b^**	***df***	***p***
Gender							
Male	48	76.2	34	65.4	1.140	1	0.286
Female	15	23.8	18	34.6			
Education Level							
Primary	22	34.9	11	21.2	2.009	1	0.156
Secondary	41	65.1	41	78.8			
Birth Place							
Hong Kong	48	84.2	37	71.2	2.724	2	0.256
Mainland China	8	14.0	13	25.0			
Others	1	1.8	2	3.8			
Religion							
None	37	67.3	29	55.8	7.725	4	0.102
Christianity	12	21.8	15	28.8			
Catholic	0	0.0	5	9.6			
Buddhism	5	9.1	3	5.8			
Others	1	1.8	0	0.0			
Family Monthly Income in USD ^c^							
>3200	14	35.0	9	22.0	3.218	3	0.359
2000–3200	13	32.5	15	36.6			
1000–2000	11	27.5	11	26.8			
<1000	2	5.0	6	14.6			
Receiving Government Subsidy							
No	40	88.9	41	83.7	0.187	1	0.665
Yes	5	11.1	8	16.3			
Paternal Education Attainment							
Illiterate	0	0.0	1	2.0	3.021	4	0.554
Primary School	11	23.4	8	16.0			
Lower Secondary	10	21.3	16	32.0			
Upper Secondary	16	34.0	17	34.0			
Post-Secondary	10	21.3	8	16.0			
Maternal Education Attainment							
Primary School	12	26.1	11	22.0	0.588	3	0.899
Lower Secondary	13	28.3	16	32.0			
Upper Secondary	17	37.0	17	34.0			
Post-Secondary	4	8.7	6	12.0			
Parents Married							
No	9	17.0	9	18.4	0.000	1	1.000
Yes	44	83.0	40	81.6			
Parents Living Together							
No	14	26.4	11	22.4	0.055	1	0.814
Yes	39	73.6	38	77.6			
Parents Divorced							
No	46	86.8	42	85.7	0.000	1	1.000
Yes	7	13.2	7	14.3			
Living With Both Parents							
No	15	27.3	18	34.6	0.375	1	0.540
Yes	40	72.7	34	65.4			
Living With Siblings							
No	27	49.1	22	42.3	0.260	1	0.610
Yes	28	50.9	30	57.7			
Living With Grandparents or Relatives							
No	39	70.9	44	84.6	2.152	1	0.142
Yes	16	29.1	8	15.4			

^a^ Independent sample *t*-test, 2-tailed; ^b^ Chi square test, 2-sided; ^c^ United States dollars.

**Table 2:** Estimates of anthropometrics impact in complex sample general linear model.

Primary Outcome Measures	Intervention		Control		Adjusted Difference	95% CI Lower	95% CI Upper	*p*	Covariates
	Mean	*SE*	Mean	*SE*					
Anthropometrics									
Height (m)	1.54	0.005	1.55	0.006	−0.005	−0.02	0.007	0.29	gd, pb, rb, gr, lp, ms, fp
Weight (kg)	62.67	0.36	63.23	0.35	−0.56	−1.42	0.30	0.11	gd, pb, rb, gr, lp, ms
BMI (kg/m^2^)	25.72	0.18	25.87	0.16	−0.14	−0.50	0.22	0.33	gd, pb, rb, gr, lm, ms
Skinfold thickness of triceps (mm) ^a^	25.05	0.76	25.97	0.60	−0.92	−2.46	0.63	0.15	gd, pb, rb
Subscapular skinfold thickness (mm) ^b^	27.97	0.73	29.43	0.70	−1.45	−3.81	0.91	0.13	gd, pb, rb, gr
Waist-to-hip ratio	0.89	0.007	0.89	0.006	0.002	−0.02	0.03	0.83	gd, pb, rb, mi
Body fat (%)	30.38	0.79	30.17	0.27	0.21	−1.46	1.88	0.76	gd, pb, rb, gr, ms

*SE* = standard error. CI = confidence interval. *p* = *p* value. ^a^ Average skinfold thickness of left and right triceps. ^b^ Average skinfold thickness of left and right scapulars. gd = gender; gr = grade; pb = place of birth; rb = religious belief; lp = living with parents; lm = living with maid; mi = family monthly income from employment; fp = father passed away; ms: parents married.

**Table 3:** Estimates of lifestyle health knowledge in complex sample general linear model.

Primary Outcome Measures	Intervention		Control		Adjusted Difference	95% CI Lower	95% CI Upper	*p*	Covariates
	Mean	*SE*	Mean	*SE*					
Lifestyle Health Knowledge									
Score on Food Pyramid Test	6.11	0.47	6.59	0.48	−0.47	−2.12	1.17	0.48	gd, pb, rb, lg, fp
Score on Sports Pyramid Test	3.23	0.24	1.86	0.11	1.37 ***	0.54	2.20	<0.001	gd, pb, rb
Score on Snack Choice Test	52.26	2.98	43.29	3.64	8.97 *	−1.83	19.77	0.04*	gd, pb, rb, me

*SE* = standard error. CI = confidence interval. *p* = *p* value. ^a^ Average skinfold thickness of left and right triceps. ^b^ Average skinfold thickness of left and right scapulars. gd = gender; pb = place of birth; rb = religious belief; lg = living with grandparent or relative; me = maternal education level; fp = father passed away.

**Table 4:** Estimates of self-efficacy in complex sample general linear model.

Secondary Outcome Measures	Intervention		Control		Adjusted Difference	95% CI Lower	95% CI Upper	*p*	Covariates
	Mean	SE	Mean	SE					
Self-Efficacy Nutrition Self-Efficacy Scale	14.24	0.85	13.72	0.97	0.52	−2.28	3.32	0.65	gd, pb, rb
Children’s Self-Efficacy in Peer Interaction Scale	43.57	2.95	40.28	2.68	3.29	−1.80	8.37	0.11	gd, pb, rb

SE = standard error. CI = confidence interval. *p* = *p* value. gd = gender; pb = place of birth; rb = religious belief.

**Table 5:** Estimates of psychosocial impacts in complex sample general linear model.

Secondary Outcome Measures	Intervention		Control		Adjusted Difference	95% CI Lower	95% CI Upper	*p*	Covariates
	Mean	*SE*	Mean	*SE*					
Psychosocial Well-Being Outcomes									
Quality of Life	68.56	2.79	60.61	2.47	7.95 ***	6.19	9.72	<0.001	gd, pb, rb
Self-esteem	20.24	1.26	18.12	0.98	2.13 ***	0.83	3.42	<0.001	gd, pb, rb, gr, lp, gs
Self-Figure Rating Scale	4.56	0.21	5.87	0.24	−1.30 ***	−2.09	−0.52	<0.001	gd, pb, rb, ms
Perceived Body Image Questionnaire	2.07	0.10	2.28	0.07	−0.21 **	−0.40	−0.02	0.008	gd, pb, rb
Perceived Body Shape	1.76	0.12	1.86	0.09	−0.10	−0.31	0.11	0.23	gd, pb, rb, ls

SE = standard error. CI = confidence interval. *p* = *p* value. gd = gender; pb = place of birth; rb = religious belief; lp = living with parents; gs = receiving government subsidy; ms = parents married;ls = living with sibling; ** *p* < 0.01. *** *p* < 0.001.

**Table 6:** Estimates of the social relationships among peers, parents and teachers with the students.

Secondary Outcome Measures	Intervention		Control		Adjusted Difference	95% CI Lower	95% CI Upper	*p*	Covariates
	Mean	*SE*	Mean	*SE*					
Social Relationships									
Relationship with father	0.92	0.21	0.69	0.25	0.23	−0.15	0.62	0.14	gd, pb, rb, lp, pe, gs, ms
Relationship with mother	1.05	0.19	0.85	0.10	0.21	−0.21	0.62	0.22	gd, pb, rb, lp, lt
Relationship with teachers and school nurse	1.04	0.18	0.94	0.09	0.11	−0.29	0.51	0.51	gd, pb, rb

*SE* = standard error. CI = confidence interval. *p* = *p* value. gd = gender; pb = place of birth; rb = religious belief; lp = living with parents; pe = paternal education level; gs = receiving government subsidy; ms = parents married; lt = parents living together.

**Table 7:** Estimates of students’ and their parents’ preferences of cooking methods at home in complex sample binary logistic regression.

Secondary Outcome Measures	B	*SE*	χ^2^	*df*	*p*	*OR*	95% CILower	95% CIUpper	Covariates
Cooking Methods Parents’ preferred cooking method ^a^	0.05	0.50	0.01	1	0.92	1.05	0.31	3.60	gd, pb, rb, dv
Students’ preferred cooking method ^b^	−0.22	0.21	1.10	1	0.29	0.81	0.49	1.34	gd, pb, rb

B = log odds. *SE* = standard error. χ^2^ = adjusted Wald chi squared. *df* = degrees of freedom. *p* = *p* value. *OR* = odds ratio. CI = confidence interval. ^a^ Parents’ preference cooking method is frying or deep frying. ^b^ Students’ preference cooking method is frying or deep frying. gd = gender; pb = place of birth; rb = religious belief; dv = parents divorced.
